# Herausforderungen und Handlungsempfehlungen für die VR-basierte Zusammenarbeit mit digitalen Nomad*innen und anderen Remote-Arbeitenden

**DOI:** 10.1365/s40702-021-00825-w

**Published:** 2021-12-21

**Authors:** Lennart Hofeditz, Ulrike Löffler, Clara Strathmann

**Affiliations:** Universität Duisburg-Essen, Duisburg, Deutschland

**Keywords:** Virtual Reality, Virtuelle Zusammenarbeit, Remote-Arbeit, Digitale Nomad*innen, Systematische Literaturanalyse, Experteninterviews, Virtual reality, Virtual collaboration, Remote work, Digital nomads, Systematic literature review, Expert interviews

## Abstract

Virtual Reality (VR) Technologien sind weit mehr als bloße Plattformen für Videospiele und Unterhaltung. Sie dienen Unternehmen aus unterschiedlichen Branchen als Werkzeuge zur Verbesserung der virtuellen Kommunikation und Teamarbeit. Insbesondere bei Mitarbeiter*innen, die in weit verbreiteten Teams arbeiten, kann VR dazu dienen, Probleme zu visualisieren und die Zusammenarbeit zu erleichtern. Viele Unternehmen beschäftigen jedoch nicht nur festangestellte Mitarbeiter*innen, sondern auch Freiberufler*innen und digitale Nomad*innen. Diese sind zwar oft flexibel und gut qualifiziert, jedoch stellen sie Unternehmen auch vor neue Herausforderungen, da ihr Selbstverständnis des Arbeitens oft mit den festen Strukturen der Unternehmen kollidiert. VR Technologien können dazu beitragen, die Zusammenarbeit mit Freiberufler*innen, digitalen Nomad*innen und anderen Remote-Arbeitenden zu erleichtern. Die Herausforderungen und Lösungsansätze, die mit dem Einsatz von VR verbunden sind, wurden jedoch noch nicht systematisch untersucht, obwohl VR-Technologien immer häufiger zum Einsatz kommen. Deshalb werden in diesem Beitrag die Ergebnisse einer systematischen Literaturanalyse zu den Herausforderungen und Lösungsansätzen präsentiert und mit acht Remote-arbeitenden Expert*innen diskutiert. Dazu wurden vier Kategorien von Herausforderungen ermittelt: Organisatorische, unternehmensbezogene, benutzerbezogene und technische Hindernisse. Für jede Kategorie werden entsprechende Handlungsempfehlungen bereitgestellt. Dieser Beitrag bietet erste Einblicke in die Nutzung von VR Technologien zur Verbesserung der Zusammenarbeit von digitalen Nomad*innen und anderen Remote-arbeitenden Freiberufler*innen und zeigt Unternehmen einen innovativen Ansatz auf. Die gewonnenen Erkenntnisse können ebenfalls angewendet werden, um aufkommende Herausforderungen zu identifizieren, zu vermeiden und zu adressieren. Dies verbessert die virtuelle Zusammenarbeit in Unternehmen und unter digitalen Nomad*innen und Freiberufler*innen.

## Abstract (en)

Virtual Reality (VR) technologies are much more than mere platforms for video games and entertainment. They serve as tools for companies in a variety of industries, to improve virtual communication and teamwork. Especially for employees working in widely distributed teams, VR can be used to visualize problems and facilitate collaboration. However, many companies employ not only permanent employees, but also freelancers and digital nomads. These are often flexible and well qualified, but they also pose new challenges to companies, as their self-image of working often collides with the fixed structures of the company. VR technologies can help facilitate collaboration with freelancers, digital nomads and other remote-workers. However, the challenges and solutions associated with the use of VR have not yet been systematically explored, despite the increasing use of VR technologies. Therefore, this paper presents the results of a systematic literature review on the challenges and solution approaches and discusses them with eight remote-working experts. For this purpose, four categories of challenges were identified: organizational, company-related, user-related, and technical barriers. For each category, corresponding recommendations for action are provided. This paper provides initial insights into the use of VR technologies to improve collaboration among digital nomads and other remote-working freelancers, and shows companies an innovative approach. The lessons learned can also be applied to identify, avoid, and address emerging challenges. This improves virtual collaboration in companies and among digital nomads and freelancers.

## Einleitung

„Experience Theater“ nannte der Kameramann Morten Heilig 1962 seine Erfindung, die auch als „Sensorama“ bekannt wurde. Seine Technologie ist eines der frühesten Beispiele für Multimodalität und Immersion und hat den Weg für die Idee der multisensorischen Unterhaltung geebnet. Heute haben wir nicht nur mehr tragbare Geräte, die den Eindruck erwecken, an einem anderen Ort zu sein, wie VR-Brillen, sondern auch die Idee dessen, was heute als virtuelle Realität (VR) bekannt ist, hat sich auf Bereiche jenseits von Freizeitanwendungen ausgedehnt. Dies umfasst Bereiche wie Medizin (Darnall et al. 2020) und Tourismus (Baxter 2020), aber auch Unternehmen, die die Möglichkeiten für kreative Zusammenarbeit erkannt haben (The Automotive Council 2010). Nicht nur wegen ihres Rufes, Kosten zu senken (Ivanova 2018), sondern auch wegen der Möglichkeit, Mitarbeiter, die aus der Ferne arbeiten, zu verbinden, hat VR-Zusammenarbeit an Aufmerksamkeit gewonnen. Im weiteren Verlauf dieses Beitrags sprechen wir bei VR-Zusammenarbeit auch von virtueller Kollaboration. Dieses Phänomen erweist sich als von wachsender Bedeutung, da Gruppen von Remote-Arbeitenden, wie digitale Nomad*innen und andere Freiberufler*innen in den letzten Jahren einen stetigen Zuwachs erfuhren (AND.CO 2017). Digitale Nomad*innen stellen dabei einen speziellen Typ von Remote-Arbeitenden dar, der über keinen festen Arbeitsplatz verfügt und sich meist auf Reisen befindet und währenddessen Auftragsarbeit erledigt. Mit der zunehmenden Anzahl von Menschen, die außerhalb typischer Büros arbeiten, steigt die Herausforderung, effektiv mit diesen Mitarbeiter*innen zu kommunizieren, zusammenzuarbeiten und sich mit ihnen zu vernetzen.

Hier kann der Einsatz von VR-Technologien zur virtuellen Zusammenarbeit ansetzen: Zum einen kann die örtliche Distanz überwunden werden, zum anderen lassen sich im Vergleich zu Plattformen mit weniger Dimensionen positive Effekte auf die Kreativität und die Arbeitsleistung beobachten (Alahuhta et al. 2014). Trotz der Fähigkeit von VR, einigen der Herausforderungen entgegenzuwirken, die Remote-Arbeit mit sich bringt, können diese Vorteile leicht von den Hindernissen überschattet werden, die im Hinblick auf die Implementierung und Akzeptanz überwunden werden müssen (Sagnier et al. 2020). Abgesehen von der Herausforderung, die Daten der Nutzer*innen zu schützen, können gesundheitliche Auswirkungen wie Motion Sickness auftreten, während der Umgang mit der Ablehnung der Technologie durch Nutzer*innen eine weitere Aufgabe darstellt. Speziell bezogen auf digitale Nomad*innen gibt es diesbezüglich noch keine Studien. Es ist jedoch anzunehmen, dass dort auch die technische Ausstattung in der Ferne eine weitere Herausforderung darstellt.

Die COVID-19-Pandemie hat der Fernarbeit einen weiteren Aufschwung verliehen. Da viele Menschen diese Art der Arbeit, die ihnen zunächst aufgezwungen wurde, nun für sich entdeckten, gehen langfristige Prognosen davon aus, dass die Fernarbeit nach der Pandemie nicht zurückgehen wird (Foss 2021). Insbesondere VR bietet attraktive Lösungen, da im Gegensatz zu bspw. Augmented Reality die Zusammenarbeit aus der Ferne in Echtzeit ermöglicht wird. Darüber hinaus hat sich die Technologie zur Unterstützung virtueller Arbeit rasant verbessert, was nur einer der Aspekte ist, die den Einsatz von VR heute so attraktiv machen. Wie von Gartners Hype Cycle 2017 prognostiziert, ist VR mittlerweile nicht mehr weit von der Mainstream-Akzeptanz in Unternehmen entfernt (Panetta 2017) und sollte in der Forschung entsprechend behandelt werden.

In diesem Beitrag beleuchten wir deshalb die Phänomene Remote-Arbeit und unternehmensbezogene virtuelle Zusammenarbeit zwischen Unternehmen und Freiberufler*innen und digitalen Nomad*innen mittels VR, sowie die damit verbundenen Herausforderungen und Lösungsansätze. Um die Implementierung von VR-Anwendungen für die Zusammenarbeit zu erleichtern, bieten wir eine Grundlage für das Verständnis der damit verbundenen Herausforderungen und zeigen in diesem Rahmen Lösungsansätze auf, die die Zusammenarbeit mit Remote-Arbeitenden, wie zum Beispiel digitalen Nomad*innen, verbessern können. Dazu beziehen wir uns auf die Ergebnisse einer systematischen Literaturanalyse und auf Einschätzung durch verschiedene Expert*innen aus der Unternehmenspraxis.

## Potenziale von Virtual Reality für Unternehmen

VR umfasst nicht-reale Umgebungen, die die Sinne der Teilnehmer*innen vollständig absorbieren. Mit anderen Worten umfasst VR eine Umgebung der vollständigen Immersion, in der die Person innerhalb einer vollständig künstlichen Welt interagieren kann. Immersion als eines der Schlüsselkonzepte in VR ist die objektive Leistung einer Technologie in Bezug auf die Schaffung eines sensorischen Äquivalents zu einer realen Erfahrung. Das heißt, je realistischer eine virtuelle Erfahrung aus technologischer Sicht dargestellt werden kann, desto immersiver ist das Gerät, das diese Erfahrung erzeugt.

Die Gründe, warum sich immer mehr Unternehmen auf VR als Technologietrend einstellen, sind so vielfältig wie ihre Anwendungsgebiete und unterscheiden sich je nach Branche. So hat VR zum Beispiel den Vorteil, Menschen sicher auf reale und gegebenenfalls gefährliche Situationen vorzubereiten, indem sie in einer ungefährlichen Umgebung trainieren können, wie zum Beispiel bei Soldat*innen, Feuerwehrleuten oder Pilot*innen. Der allgemeine Vorteil der Immersion und Interaktivität findet seinen Wert auch in der Bildung, wo der Lernerfolg positiv mit dem Immersionsgrad der verwendeten Technologie korreliert (Gutierrez et al. 2007). Einer der wohl überzeugendsten Vorteile aus Unternehmenssicht liegt in der Reduzierung von Kosten (Ivanova 2018). So sparte Jaguar Millionen von Pfund durch den Einsatz eines VR-Engineering- und Designstudios. Die Mitarbeiter*innen arbeiteten nur noch mit virtuellen Komponenten und Strukturen, sodass Jaguar nicht nur Zeit, sondern auch Kosten für die Herstellung physischer Prototypen einsparen konnte. Abgesehen von den Produktionskosten kann VR auch viele vor-Ort-Meetings ersetzen, sodass Kosten für Reisen oder Organisation eingespart werden können. So können Teams aus verschiedenen Städten, Ländern und Kontinenten zusammenarbeiten, während sie ein ähnliches Gefühl verspüren, als wenn sie zusammen in einem Büro in einem Konferenzraum säßen. Während argumentiert werden kann, dass reguläre Videoanrufe ohne VR den Zweck einer interkontinentalen Verbindung erfüllen könnten, zeigt die Forschung, dass die Anwendung von VR in diesem Kontext sowohl die Kommunikation als auch die Zusammenarbeit weitaus positiver beeinflussen kann (Breves 2021). So kann eine stärkere Immersion die Leistung der Arbeitenden bei bestimmten Teamaufgaben, sowie die allgemeine Kommunikationsbereitschaft der Mitarbeiter*innen untereinander verbessern. Darüber hinaus sind vielversprechende Möglichkeiten zur Integration von Remote-Mitarbeiter*innen in bestehende Teams anzunehmen. Jedoch beschäftigt sich aktuelle Forschung vorrangig mit der Betrachtung der Effekte von VR auf die Zusammenarbeit bei schon bestehenden Teams (Breves 2021). Die Arbeitnehmerschaft in heutigen Unternehmen besteht jedoch immer mehr aus Freiberufler*innen und remote-arbeitenden Personen (Sako 2021). Eine besonders unabhängige Gruppe dieser remote-arbeitenden Personen werden auch als digitale Nomad*innen bezeichnet (Richter und Richter 2020). Wir beschreiben diese zusammenarbeitenden Gruppen aus festangestellten und nicht festangestellten remote-arbeitenden Personen im Folgenden als heterogene Teams.

## Verbesserung der Zusammenarbeit mit Remote-Arbeitenden durch Virtual Reality

### Herausforderungen der Zusammenarbeit mit digitalen Nomad*innen und Freiberufler*innen

Wenn man an die*den durchschnittlichen Arbeitende*n denkt, ruft das bei vielen Menschen ein Bild einer Person hervor, die in einem Büro an einem festen Arbeitsplatz bei einem bestimmten Unternehmen vor einem Computer mit einer Tasse Kaffee sitzt. Betrachtet man jedoch die jüngsten Statistiken, so ist diese Art des Arbeitens weit weniger üblich geworden. Laptop und Kaffee mögen zwar geblieben sein, aber eine wachsende Zahl von Menschen arbeitete auch schon vor der Covid-19 Pandemie von zu Hause. Laut GlobalWorkplaceAnalytics.com (2020) ist die regelmäßige Arbeit zu Hause in den USA zwischen 2005 und März 2020 um 173 % gestiegen, Selbstständige nicht mitgerechnet. Zwei Personengruppen werden dabei häufig genannt, wenn es um Remote-Arbeit geht: Freiberufler*innen und digitale Nomad*innen. Erstere werden als individuelle, zeitlich begrenzte Auftragnehmer*innen definiert, die ihre eigenen Fähigkeiten einsetzen, um Kund*innen zu betreuen (Kazi et al. 2014). Eine Umfrage ergab, dass 86 % der meist amerikanischen Freiberufler*innen typischerweise von zu Hause oder anderen externen Orten wie Cafés (40 %) arbeiten, während nur 25 % regelmäßig in die Büros ihrer Kund*innen fahren (AND.CO 2017). Im Vergleich dazu ist die Remote-Arbeit ein explizit spezifiziertes Kernmerkmal von digitalen Nomad*innen, die als Menschen definiert werden, die Informations- und Kommunikationstechnologien nutzen, um von entfernten Orten aus zu arbeiten, während sie meist aus Lifestyle-Gründen reisen (Reichenberger 2018). Die Kerncharakteristika der Freiberufler*innen und digitalen Nomad*innen gleichen sich somit in vielen Punkten; Beide arbeiten eher nicht am klassischen Arbeitsplatz in einem Büro eines Unternehmens. Digitale Nomad*innen arbeiten hier in der Regel von unterwegs (z. B. in Coworking Spaces oder in Cafés) und sind durch klassische Führungskräfte schwierig zu managen (Frick und Marx 2021), Freiberufler*innen arbeiten ebenfalls gelegentlich von unterwegs, arbeiten jedoch meist von zu Hause (und somit immer am selben Ort). Bezüglich des Arbeitsverhältnisses lässt sich des Weiteren differenzieren, dass Freiberufler*innen über kein festes Arbeitsverhältnis verfügen, sondern auftragsweise arbeiten; Digitale Nomad*innen können sowohl einen festen Arbeitsvertrag und somit eine direkte Führungsperson haben als auch ein auftragsbasiertes Vertragsverhältnis (Frick und Marx 2021). Digitale Nomad*innen sowie Freiberufler*innen haben zudem gemeinsam, dass Autonomie und Unabhängigkeit für sie einen sehr hohen Stellenwert einnehmen.

Reichenberger (2018) kategorisiert vier verschiedene Stufen des digitalen Nomad*innentums, die schrittweise aufeinander aufbauen: Level 0‑Nomad*innen zeichnen sich dadurch aus, dass sie durch das Arbeiten in einer Online-Umgebung ortsunabhängig sind, was sich in Level 1 auf das nicht konsequente Arbeiten in einem bestimmten Büroraum erstreckt. Erst Level 2 bringt den Aspekt des Reisens während der Arbeit mit sich, während eine Person als digitale*r Nomade*in der Stufe 3 gilt, wenn kein fester Wohnsitz mehr besteht. Vor allem Level 2 und 3 verstehen sich als digitale Nomad*innen, da die unteren Level auch Merkmale enthalten, die in anderen Bereichen der Remote-Arbeit zu finden sind.

Ein Blick auf die Zahlen zeigt, dass diese beiden Modelle immer beliebter werden: Während die Zahl der Menschen, die sich als digitale Nomad*innen bezeichnen, in den USA von 2019 (7,3 Mio.) bis 2020 (10,9 Mio.) um 49 % gestiegen ist (MBO Partners 2020), hat sich die Zahl der Freiberufler*innen innerhalb von fünf Jahren von 53 auf 57 Mio. erhöht, was bedeutet, dass 2019 insgesamt 35 % der amerikanischen Arbeitnehmer*innen freiberuflich tätig waren (AND.CO 2017). Die häufigsten Bereiche, in denen Freiberufler*innen tätig sind, wurden als „kreativ“ (33 %) beschrieben, gefolgt von „Beratung/professionelle Dienstleistungen“ (21 %) und „Schreiben, Journalismus oder Content Services“ (17 %). Darüber hinaus ist eine der Begleiterscheinungen der COVID-19-Pandemie eine Zunahme der Remote-Arbeit, die langfristig die globale Arbeitsmoral beeinflussen soll (Bloom 2020; Foss 2021) und den Bedarf an wissenschaftlichen Arbeiten in diesem Bereich unterstreicht. Aufgrund der oben genannten Daten scheint es weiterhin sinnvoll, sowohl Freiberufler*innen als auch digitale Nomad*innen als wichtige Bindeglieder in der Fernarbeitsforschung anzunehmen.

Trotz der Vorteile der Fernarbeit, wie Flexibilität oder Autonomie, bringt diese Art der Arbeit auch gewisse Herausforderungen mit sich. So können die fehlende persönliche Betreuung, der erschwerte Zugang zu Informationen, die soziale Isolation und Ablenkungen zu Hause Hürden für die Zusammenarbeit mit diesen heterogenen Gruppen darstellen. Ein Problem entsteht durch die reduzierte Face-to-Face-Interaktion, die sowohl für die*den Vorgesetzte*n wichtig ist, um sicherzustellen, dass die Arbeit erledigt wird, als auch für Mitarbeiter*innen im Hinblick auf die Unterstützung durch die Führungskraft. Der Zugang zu Informationen kann dann problematisch werden, wenn Remote-Mitarbeiter*innen organisatorische Schwierigkeiten haben, benötigten Input von Kolleg*innen zu erhalten, während die soziale Isolation ein eher psychologisches Phänomen beschreibt. So kann als eine der häufigsten Beschwerden das Gefühl der Einsamkeit, das auf lange Sicht sogar die Absicht von Mitarbeiter*innen, das Unternehmen zu verlassen, verstärken kann, genannt werden. Schließlich stellen die Ablenkungen zu Hause oder in einem Café eine weitere Herausforderung dar, da Störgeräusche auftreten können und die Kombination von Freizeit und Arbeit in der gleichen Umgebung bewältigt werden müssen. Betrachtet man einige der Vorteile, die VR bietet, wie z. B. verbesserte Kommunikations- und Kollaborationsprozesse durch Interaktivität und Immersion, so kann angenommen werden, dass diese Technologien eine wertvolle Möglichkeit bieten können, mit den Remote-Mitarbeiter*innen wie digitalen Nomad*innen in Kontakt zu treten und sie in den Arbeitskontext des Unternehmens einzugliedern. Jedoch ist es für Unternehmen herausfordernd, VR in der Praxis einzusetzen, da viele vor Kosten, fehlender Akzeptanz oder anderen Hürden zurückschrecken. Deshalb präsentieren wir in diesem Beitrag einige Lösungsansätze für bestimmte Herausforderungen.

### Vorteile der Virtual Reality-Nutzung für die digitale Zusammenarbeit

**Abb. 1:**
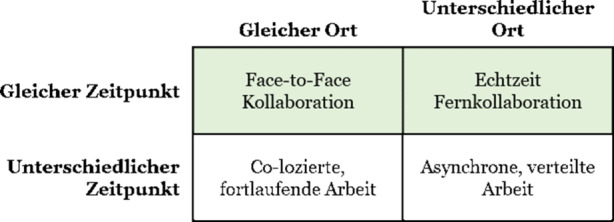
Mixed Presence Groupware in Ort/Zeit-Matrix (Regenbrecht et al. 2006, S. 98)

Die einzige Möglichkeit der synchronen Zusammenarbeit mit Kolleg*innen, die sich nicht im selben Büro befinden, ist die Nutzung digitaler und virtueller Technologien. Regenbrecht et al. (2006) geben einen Überblick über zeit- und ortsabhängige Kollaborationstypen, indem sie eine Groupware-Matrix erstellen, die vier verschiedene Kombinationen von Ort und Zeit umfasst (Abb. 1). Den Autoren zufolge fällt die virtuelle Teamarbeit mittels VR-Technologien hauptsächlich in die Kategorie „different place, same time“, die sie als Echtzeit-Fernkollaboration bezeichnen. Systeme, die diese Art der synchronen Zusammenarbeit unterstützen, bezeichnen sie als Mixed Presence Groupware (MPG). Die gegebene Definition solcher Systeme als „ein gemeinsamer visueller Arbeitsraum [der] auch verteilte Teilnehmer in Echtzeit unterstützt“ (S. 97) dient als Orientierung dafür, welchen Zweck ein VR-Kollaborationssystem erfüllen soll.

Regenbrecht et al. (2006) weisen auch darauf hin, dass sich Fernarbeit negativ auf die kreative Leistung auswirken kann, wenn ein solches Gefühl der Face-to-Face-Interaktion, vermittelt durch MPGs, fehlt. Tatsächlich hat die Forschung gezeigt, dass virtuelle Welten eine Reihe von Merkmalen bieten können, die die Teamkreativität fördern können, wie z. B. Immersion, Multimodalität und die mögliche Nutzung unterstützender Tools. Mit Blick auf die Umfrageergebnisse von AND.CO (2017), die besagen, dass zumindest ein Großteil der Freiberufler*innen in kreativen Branchen arbeitet, scheint sich der Einsatz von VR-Technologien für die Zusammenarbeit als potenziell effektiv zu erweisen, um die kreative Energie von Remote-Arbeitenden zu unterstützen.

Mittlerweile gibt es verschiedene VR-Anwendungen für Kommunikations- und Kollaborationszwecke. Eine davon ist Spatial^1^. Dieses bietet eine Umgebung, in der Arbeitende mit Kolleg*innen interagieren können, die durch hologrammartige Avatare dargestellt werden und durch das Hochladen eines Fotos erstellt werden. Dadurch kann die Software eine 3D-Version einer Person erzeugen, die über ein echtes Gesicht, einen bewegungssensitiven Kopf sowie Lippenbewegungen verfügt, die auf die Stimme des Sprechers abgestimmt sind. Die technische Grundlage dafür ist durch ein VR-Headset gegeben, wobei die günstigsten Modelle mittlerweile schon ab 300 Dollar verfügbar sind^2^. Auch wenn nicht alle Teammitglieder*innen über die entsprechenden Geräte verfügen, so besteht bei vielen Anwendungen die Möglichkeit, mittels der Webcam eines Laptops an VR Meetings teilzunehmen. Einige neuartigere VR-Anwendungen bieten in ihren virtuellen Umgebungen sprachgesteuerte 3D-Webbrowser an, die Objekte in den gemeinsamen Raum übertragen können, ebenso wie klassische Whiteboards oder Screensharing. Insbesondere Spatial wurde als „außergewöhnlich“ und „disruptiv“ (Fink 2018) und als Einführung in „die Zukunft von Remote-Arbeit und Meetings“ (Horwitz 2020) beschrieben und wird bereits von großen Unternehmen wie Mattel, Ford und der Telekom eingesetzt.

## Datengrundlage für identifizierte Herausforderungen und Handlungsempfehlungen

Während die virtuelle Arbeit bei heterogenen Teams mittels VR-Technologien die Möglichkeit bietet, kreativ mit anderen Kolleg*innen und Remote-Arbeitenden wie digitalen Nomad*innen zu interagieren, birgt die Implementierung und Nutzung von VR im Unternehmenskontext auch gewisse Hindernisse, die es zu überwinden gilt, um eine erfolgreiche Nutzung zu gewährleisten. Insbesondere für die Zusammenarbeit mit digitalen Nomad*innen oder anderen Freiberufler*innen kommen zusätzlich zu den Vorteilen der VR Nutzung einige Herausforderungen auf Unternehmen zu. Um Herausforderungen, aber auch Lösungsansätze für die Nutzung von VR in der Zusammenarbeit mit Remote-Arbeitenden, digitalen Nomad*innen oder Freiberufler*innen zu identifizieren, präsentieren wir in diesem Beitrag die Ergebnisse eines systematischen Literaturreviews welches insgesamt 245 Forschungsartikel einschließt. Zusätzlich haben wir acht Expert*innen aus der Praxis interviewt und sie gebeten, Herausforderungen und Lösungsansätze zu beurteilen. Bei den Expert*innen handelte es sich um Remote-Arbeitende mit VR-Erfahrung aus verschiedenen technologienahen Branchen. Alle Interviewpartner*innen gaben zudem an, von zu Hause oder von unterwegs zu arbeiten. Somit handelte es sich zwar per Definition um keine reinen digitalen Nomad*innen, sondern vielmehr um Angestellte und Freiberufler*innen, die gelegentlich von unterwegs arbeiten. Wie jedoch bereits beschrieben, sind beide Gruppen eng miteinander verwoben, wobei remote-arbeitende Freiberufler*innen und digitale Nomad*innen eine große gemeinsame Schnittmenge aufweisen.

Die durch die systematische Stichwortsuche identifizierten Veröffentlichungen wurden alle zwischen 2002 und 2021 publiziert. Häufig wurde VR als neue Möglichkeit der Kommunikation und Zusammenarbeit genannt, die aber noch nicht weit verbreitet ist. Fast alle identifizierten Arbeiten sprechen von sogenannten virtuellen Organisationen oder Unternehmen, in denen Freiberufler*innen, digitale Nomad*innen oder Selbstständige arbeiten. Einige Beiträge beschäftigten sich länderspezifisch mit der Nutzung von VR. VR wurde oft als Zukunftslösung beschrieben, nicht als aktueller Zustand. Der genaue Analyseprozess bei der systematischen Literatursuche wird in Anhang A zusammengefasst.

## Herausforderungen für die VR-unterstützte Zusammenarbeit mit Remote-Arbeitenden

In Tab. 1 beschreiben wir die aus der Literatur abgeleiteten Herausforderungen für die VR-unterstützte Zusammenarbeit von Unternehmen mit Remote-Arbeitenden und insbesondere digitalen Nomad*innen. Insgesamt konnten wir diese aus 25 Literaturquellen ableiten. Die Literaturquellen sind in Anhang B aufgeführt. Es zeichneten sich vier Hauptkategorien von Herausforderungen ab: Herausforderungen für Führungskräfte, unternehmensspezifische Herausforderungen, benutzerbezogene Herausforderungen und technische Herausforderungen.

**Tab. 1:** Herausforderungen bei der Nutzung von VR für die Zusammenarbeit mit Remote-Arbeitenden sowie digitalen Nomad*innen

Kategorie	Von Unternehmen zu adressierende Herausforderungen
Herausforderungen für Führungskräfte	Schulungs- und Lernphasen ermöglichen
	Unterschiedliche Erfahrungen, Alter, Fähigkeiten, Interessen, Ziele, Kulturen oder organisatorischen Hintergrund berücksichtigen
	Änderung organisatorischer Strukturen berücksichtigen
	Akzeptanz für Veränderungen auf organisatorischer Ebene schaffen
	Nicht versuchen, durch VR die Face-to-Face-Kommunikation vollständig zu ersetzen, sondern gezielte Anwendungsfälle identifizieren
	Alle potenziellen Anwender*innen in die Einführung einer VR-Anwendung mit einbeziehen
	Der potenziellen Destruktion der bestehenden sozialen Ordnung entgegenwirken
	Neue Wege des Informations- und Wissensaustauschs identifizieren
	Neue Kontroll- und Anreizaspekte und Leistungsmessung identifizieren
Unternehmensspezifische Herausforderungen	Zeit- und Kosten der Implementierung frühzeitig berücksichtigen
	Spezielle Technologie und Software frühzeitig bereitstellen
	Nutzen für kleine Betriebe genau überprüfen
	Neue geeignete Partnerunternehmen und Dienstleister identifizieren
	Industriestandards und -richtlinien unter Berücksichtigung von Datenschutzbelangen neu definieren
Nutzerbezogene Herausforderungen	Work-Life-Balance der Arbeitenden nicht gefährden
	Zeitaufwand der Einrichtung der Nutzer*innen (z. B. um einen Avatar zu erstellen und zu lernen, wie man partizipiert und interagiert) berücksichtigen
	Den richtigen Komplexitätsgrad für die gewünschten Ziele identifizieren (Einstellungsmöglichkeiten, Controller, Basisstationen, Computer, sonstiges Equipment)
	Internet-Stress gezielt vermeiden (Informations- und Kontaktüberlastung, Erreichbarkeit, Wahrnehmung von Zeit und Raum)
	Das Navigieren und Interagieren in der virtuellen Welt nicht zu ablenkend gestalten, um das eigentliche Ziel der Zusammenarbeit zu fokussieren
	Technologieakzeptanz und breitflächige Adoption unter Nutzer*innen fördern
	Motion Sickness durch bestimmte Designtechniken vorbeugen (z. B. durch Teleportbewegungen, anstelle von fortlaufenden Bewegungen durch die VR-Welten)
	Engagement der Benutzer*innen, Motivation, persönliches Interesse und Klarheit über das Ziel identifizieren
	Bewusstsein für die Verfügbarkeit, die Aktivitäten und den Fortschritt der Anderen im Arbeitsprozess visualisieren
	Neue Kompetenzen im Beziehungsmanagement identifizieren
Technische Herausforderungen	Usability-Probleme und veraltete Technik vermeiden
	Integration von neuen Technologien in alte Systeme vermeiden
	Datensicherheit bei VR-Anwendungen überprüfen
	Leichte Headsets anstelle von komplexen Lösungen verwenden
	Aufgabenspezifische Headsets und Controller nutzen

Herausforderungen für Führungskräfte beschreiben Barrieren, die das Management- und Organisationssystem in einem Unternehmen betreffen. Zum Beispiel müssen organisatorische Strukturen angepasst werden. Unter unternehmensspezifische Herausforderungen werden Aufgaben gruppiert, die auf Unternehmensebene anfallen. Zum Beispiel müssen Industriestandards innerhalb und zwischen Unternehmen angepasst werden. Diese Herausforderungen müssen auf Unternehmensebene angegangen werden, nicht auf Abteilungsebene, wie bei den Herausforderungen für Führungskräfte. Benutzerbezogene Herausforderungen befassen sich mit nutzerspezifischen Problemen, wie z. B. Motion Sickness, die bei der Nutzung von VR auftreten kann und möglicherweise zu Kopfschmerzen, Erbrechen oder allgemeinem Unwohlsein führt. Die letzte Gruppe der identifizierten Herausforderungen adressiert technische Probleme, die entstehen können, zum Beispiel die Tatsache, dass VR-Headsets derzeit oft noch umständlich und unkomfortabel zu bedienen sind.

In allen Kategorien zeichneten sich bestimmte Herausforderungen ab, die in der Literatur häufig genannt und als gravierend betitelt wurden. Bei den organisatorischen Herausforderungen stehen beispielsweise viele Unternehmen vor dem Problem, dass die Mitarbeiter*innen unterschiedliche Erfahrungs- und Motivationsniveaus haben und somit eine angepasste Einarbeitungsphase für die Mitarbeiter*innen notwendig ist. Bei den unternehmensbezogenen Herausforderungen stellte sich kein Aspekt als besonders schwerwiegend heraus. Dennoch kann festgehalten werden, dass der Implementierungsaufwand der VR-Technologie oft hoch ist und spezielle Programmierkenntnisse erforderlich sind, um sie umzusetzen. Bei den nutzerbezogenen Herausforderungen, gibt es viele häufig genannte Herausforderungen, wie z. B. die Sicherstellung der Work-Life-Balance für digitale Nomad*innen oder Freiberufler*innen, die mit der VR-Nutzung einhergeht. VR für Kollaborationszwecke kann schnell komplex und zeitaufwendig bei der Einführung sein, wie z. B. bei der Erstellung eines personalisierten Avatars, der die eigene Person adäquat repräsentiert. Als letzte Kategorie kann der aktuelle Stand der Technik als die größte Herausforderung gesehen werden. VR-Systeme können teilweise Probleme bei der Benutzerfreundlichkeit aufweisen oder wiegen oft noch schwer auf dem Kopf. Auch sind viele Lösungen kabelgebunden oder erfordern zusätzliche Basisstationen. Einer der befragten Expert*innen empfiehlt bei aktuellen VR-Technologien:

[…] nicht mehr als eine Stunde oder zwei wirklich produktiv voll involviert in VR zu arbeiten.

(Remote Arbeitender Softwareentwickler mit Erfahrung im Bereich VR)

## Einschätzungen und Handlungsempfehlungen für die VR-unterstützte Zusammenarbeit mit digitalen Nomad*innen und anderen Remote-Arbeitenden

Im Folgenden beschreiben wir Handlungsempfehlungen für Unternehmen. Diese entstanden aus einer Analyse der Interviewmaterialien, nachdem wir die Interviewpartner*innen nach einer Einschätzung der Herausforderungen für die VR-unterstützte Zusammenarbeit befragt hatten. Die Handlungsempfehlungen werden in Tab. 2 aufgeführt.

**Tab. 2:** Handlungsempfehlungen zur Stärkung der Zusammenarbeit mit Remote-Arbeitenden

Kategorie	Handlungsempfehlungen für Unternehmen	Beispiele
Führungskräfte	1) Führungskräfte in Unternehmen sollten einige konkrete Ziele und Aufgaben für ihre Teams definieren, bei denen durch VR-Technologien die Zusammenarbeit mit digitalen Nomad*innen, Freiberufler*innen und Remote-Arbeitenden verbessert werden kann. Dabei sollte es sich vermehrt um kollaborative und kreative Aufgaben und Ziele handeln, um das Vertrauen innerhalb von heterogenen Teams zu stärken	(Design Thinking‑) Workshops zur Problemlösung Brainstorming zur Ideengenerierung Soziale Events zur Stärkung des Teamgeistes
Unternehmen	2) Unternehmen sollten zunächst den Mehrwehrt des Einsatzes von VR-Technologien zur Stärkung der Zusammenarbeit mit Remote-Arbeitenden (z. B. digitalen Nomad*innen) für ihr Unternehmen prüfen und gegebenenfalls geeignete Partner identifizieren, die die Einführung begleiten können. Unternehmensinterne Richtlinien und Bestimmungen sollten vor dem Einsatz geprüft werden, um Problemen beim Datenschutz vorzubeugen	Prüfen, ob Arbeitnehmer*innen Technologieaffinität aufweisen
		Zusammenarbeit mit Forschungsinstituten und VR-Entwicklerstudios prüfen
		DSGVO-Konformität bei VR-Nutzung prüfen
Nutzer*innen/Mitarbeiter*innen	3) Beim Einführungsprozess von VR-Technologien zur Unterstützung der Zusammenarbeit von heterogenen Teams sollten sowohl Remote-Arbeitende Personen wie digitale Nomad*innen als auch vor Ort arbeitende Angestellte involviert sein, sodass individuelle Bedürfnisse wie bestimmte Designelemente berücksichtig werden können. Wir empfehlen zunächst eine freiwillige Testphase mit Early-Adoptern, in welcher Aufgaben und Interaktionen getestet werden	Befragungen zu Herausforderungen bei der Zusammenarbeit und zur Nutzungsbereitschaft von VR-Technologien
		Technologieaffine Personen als Early-Adopter auswählen
		Erfragen, welche Designelemente (wie z. B. ein bestimmter Grafikstil) gewünscht sind
Technik	4) Zur Verbesserung der Zusammenarbeit in heterogenen Teams, sollten mobile und kompakte VR-Technologien verwendet werden, um es auch digitalen Nomad*innen oder anderen Remote-Arbeitenden zu ermöglichen, VR ortsunabhängig zu nutzen. Wir empfehlen jedoch, nicht auf Smartphone-basierte VR-Lösungen zurückzugreifen, da diese die Interaktionsmöglichkeiten einschränken. Stattdessen sollten Standalone-VR-Headsets verwendet werden	Prüfung der Verwendung von leichten Standalone-VR-Headsets (z. B. Oculus Quest 2)
		Prüfung der Verwendung einer kabellosen Technologie
		Testen von Handtracking und Controllern
		Vermeidung von Usability-Problemen durch die Verwendung schon etablierter VR-Applikationen (z. B. Spatial VR)

Um die Herausforderungen für Führungskräfte zu meistern ist es wichtig, VR als zusätzlichen Ansatz zur Zusammenarbeit in Kombination mit konservativeren Kollaborationsmethoden, wie Video- oder Telefonkonferenzen zu kombinieren. Darüber hinaus sollte auch die Erfahrung und das Qualifikationsniveau der Mitarbeiter*innenberücksichtigt werden, z. B. durch Anpassung der Dauer der Einarbeitungsphase. Eine weitere Möglichkeit ist es, die Einführung von VR zur Zusammenarbeit mit digitalen Nomad*innen und Remote-Arbeitenden informell und nicht verpflichtend zu gestalten und eine längere Startphase zuzulassen. Hier könnte es auch helfen, die Einführungsphase zuerst mit sogenannten Early-Adoptern oder technikaffinen Menschen zu beginnen. Insgesamt müssen die Organisations- und Führungsstrukturen dafür jedoch angepasst werden, um die oben genannten Herausforderungen zu bewältigen.

Zur Adressierung der Nutzerbezogenen Herausforderungen bieten sich ebenfalls einige Möglichkeiten an. Auf Grundlage unserer Ergebnisse empfehlen wir, ein Mentoring-System anzubieten, um die Nutzung, aber vor allem den Start mit der Technologie zu erleichtern. Avatare haben zudem einen großen Einfluss darauf, wie Menschen VR-Technologien wahrnehmen und mit ihnen interagieren. Aus diesem Grund schlagen wir vor, eine Personalisierbarkeit von VR-Avataren für die Nutzer*innen bereitzustellen. Die Möglichkeit für Mitarbeitende, ihren Avatar anzupassen individuell zu gestalten, diesen mit bestimmten Accessoires auszustatten oder das Gesicht real aussehen zu lassen, erhöht die emotionale Bindung, die Akzeptanz, die Identifikation und stärkt den Teamzusammenhalt. Im Allgemeinen empfehlen wir, dass sich die Aufmerksamkeit des Managements voranging auf den Nutzenden und zweitrangig auf die Technologie fokussieren sollte. Gerade zu Beginn eines Einführungsprozesses von VR-Technologien müssen Fehler in Kauf genommen und vom Management akzeptiert werden, ohne, dass die Nutzung direkt in Frage gestellt wird. Ältere Mitarbeitende benötigen bei der Einführung besonders Unterstützung und in der Regel eine längere Zeitspanne zur Akzeptanz. Eine weitere Möglichkeit, die allgemeine Akzeptanz gegenüber VR-Technologien und die Benutzerfreundlichkeit zu erhöhen, ist die Vorbereitung und Vermittlung des Einsatzes von VR bereits in Ausbildungen, Schulungen und Fortbildungen.

Um den technischen Herausforderungen zu begegnen, sollte die VR-Ausrüstung standardisiert werden. Das bedeutet, dass nicht einige wenige exklusive und experimentelle Technologien genutzt werden, sondern mobile und kostengünstige Produkte. So können verschiedene Unternehmen, aber auch digitale Nomad*innen und Remote-Arbeitende, leichter zusammenarbeiten und von den positiven Effekten der sozialen Präsenz und Immersion für die Zusammenarbeit und den Teamzusammenhalt profitieren.

Vielen Leute, die nicht aus dem Bereich kommen ist es nicht wichtig, […] die neueste Grafik zu haben, sondern ihnen ist gerade das Handling wichtig. Das ist auch die Erfahrung mit unseren Kunden. Sie nehmen meist lieber ein mobiles Headset. Für die Darstellungsqualität, im Sinne von Computing (bedeutet es), das Ganze ist graphisch einfacher dargestellt, aber (die Kunden) haben es halt gern einfach. Convenience ist wichtig, sprich: man kann so ein Gerät mitnehmen. Das ist auch meine Erfahrung, ich benutze das mobile Headset, wie die Quest deutlich öfter, als die Oculus Rift oder PC-basierte Headsets, weil es Standalone-Geräte sind, die man einfach anschaltet und sie funktionieren und man schaltet es ab und es ist wieder aus. Es ist kein großer Aufwand dahinter.

(Remote Arbeitender Softwareentwickler für Virtual- und Augmented Reality-Anwendungen)

Das bedeutet allerdings nicht, dass Unternehmen unbedingt die kostengünstigsten Lösungen wählen sollten (z. B. Google Cardboard), da dort die Immersion durch niedrige Auflösung und mangelnde Interaktionsmöglichkeiten verhältnismäßig niedrig ausfällt. Ein Softwareentwickler sagte darüber folgendes:

Auf technischer Sicht, würde ich mir wünschen, dass die Auflösung noch angezogen werden kann. Dass man Dinge auf gewisser Distanz noch klar erkennen kann. Dann kann ich mir schon vorstellen, dass es einen bestimmten Monitor oder Schreibtisch mit der Zeit ersetzen kann.

(Remote-Arbeitender Softwareentwickler mit VR-Erfahrung)

Darüber hinaus sollten verschiedene Eingabegeräte angeboten werden, um persönlichen Vorlieben oder aufgabenabhängigen Anforderungen gerecht zu werden. Aktuelle VR-Lösungen bieten dabei sowohl Controller mit verschiedenen Freiheitsgraden als auch die Unterstützung von Handgesten.

## Fazit

Dieser Beitrag vermittelt einen praktischen Überblick über Herausforderungen und Lösungsansätze im Rahmen der virtuellen Zusammenarbeit mit VR zwischen Unternehmen und digitalen Nomad*innen und anderen Freiberufler*innen, die remote arbeiten. Konkret empfehlen wir Unternehmen ein Schulungs- oder Mentoring-System zu implementieren, um Aufgaben für den Einsatz von VR-Technologien zur Unterstützung der Teamarbeit von heterogenen Teams zu identifizieren und Anwender*innen im Einführungsprozess zu unterstützen. Es könnten speziell mobile Standalone-Headsets, wie die Oculus Quest 2 zum Einsatz kommen, um einen möglichst unabhängigen Einsatz zu ermöglichen. Auch können Unternehmen, die bereits den Einsatz von VR-Technologien in der Vergangenheit getestet haben, die Erkenntnisse aus diesem Beitrag verwenden, um auf bestehende Erfahrungen aufzubauen. Viele der Barrieren und Herausforderungen der Nutzung von VR-Technologien, die noch vor einigen Jahren die Nutzungstauglichkeit schmälerten, konnten in den letzten Generationen überwunden werden. So lohnt es sich in jedem Fall die Potenziale und möglichen Einsatzgebiete, insbesondere in heterogenen Teams, die mit digitalen Nomad*innen oder anderen Remote-Arbeitenden interagieren, neu zu bewerten. Das Management innerhalb eines Unternehmens kann unsere Erkenntnisse nutzen, um sich auf noch bestehende Herausforderungen vorzubereiten oder um schon bestehende Prozesse für die virtuelle Zusammenarbeit zu verbessern. Insgesamt sind aktuelle VR-Technologien jedoch auch noch verbesserungswürdig. So könnte vor allem das Field-of-View – also das Sichtfeld der Anwender*innen – und die Auflösung weiter verbessert werden. Des Weiteren wiegen die Brillen immer noch verhältnismäßig schwer auf dem Gesicht, sodass der Tragekomfort verbessert werden muss, um eine längere Verwendung zu gewährleisten.

Diese Kategorisierung und Bewertung ist die erste umfassende Übersicht über die Herausforderungen und Lösungsansätze für Unternehmen in Bezug auf die Nutzung und Anwendung von VR-Kollaborationssystemen in Kontext von Remote-Arbeitenden und digitalen Nomad*innen. Mit der vorliegenden Arbeit wurde eine Grundlage für die weitere Forschung geschaffen – insbesondere für die Wirtschaftsinformatik – um sich auf weitere Faktoren und Forschungsbereiche spezialisieren zu können. Die Ergebnisse zeigen auch, dass die aktuelle Forschung die Virtualisierung von Kollaborationsprozessen insbesondere durch VR-Technologien nicht vollständig abdeckt, da sie sich auf technische und nutzerbezogene Faktoren konzentriert, nicht aber auf organisatorische und unternehmerische Faktoren. Für die Wirtschaftsinformatik bedeutet dies, dass weitere Faktoren identifiziert werden müssen, unter der Annahme, dass diese Faktoren auch in anderen Kontexten wichtig sind. Aus den Ergebnissen ergibt sich neuer Forschungsbedarf, der sich mit anderen Theorien wie Technologieakzeptanz oder Innovationsdiffusion in Bezug auf die Nutzung und Anwendung von VR-Kollaborationssystemen beschäftigen kann. So sollte zukünftige Forschung die Verwendung von VR-Technologien zur Stärkung der Zusammenarbeit von heterogenen Teams in konkreten Anwendungsszenarien (z. B. Workshops oder Brainstorming-Sessions) begleiten. Auch sollte die Verwendung von VR-Technologien in verschiedenen Branchen und Abteilungen erforscht und verglichen werden, um herauszufinden, für welche Zielgruppen sich der Einsatz besonders eignet. Dabei sollten auch die Potenziale von VR – insbesondere im Hinblick auf die Zusammenarbeit von heterogenen Teams – genauer herausgestellt werden. Außerdem sollte zukünftige Forschung besonders geeignete Designelemente identifizieren, die den Austausch und die Zusammenarbeit in VR anregen. Methodisch bieten sich somit Design Science Research Studien, Befragungen von Mitarbeiter*innen, Interviewstudien mit digitalen Nomad*innen oder Case Studies mit verschiedenen Unternehmen an.

## Figures and Tables

**Abb. 2:**
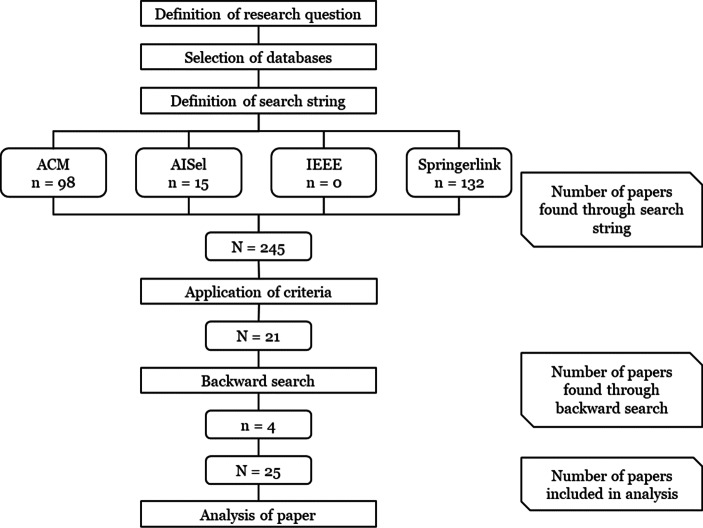
Vorgehen und Analyseschritte für das systematische Literaturreview

## Anhang

### A) Methode des systematischen Literaturreviews

Eine erste, kurze Durchsicht der Literatur ohne definierte Stichworte ergab, dass ein allgemeiner Überblick über die Herausforderungen für Unternehmen, die mit digitalen Nomad*innen und Freiberufler*innen unter Verwendung von Virtual Reality arbeiten, fehlt. Das Hauptziel dieser Recherche war die Überprüfung und Kategorisierung von Herausforderungen, Problemen und Risiken bei der Arbeit mit Virtual-Reality-Systemen. In Anlehnung an vom Brocke et al. (2015) wurde der Suchumfang definiert, um die Grundzüge der Literaturrecherche festzulegen, sichtbar in Tab. 3. Die fettgedruckten Begriffe zeigen unseren gewählten Ansatz, während die anderen Begriffe Alternativen bei der Definition des Suchumfangs darstellen.

**Tab. 3:** Auswahl der Analyseschritte

Prozess	*Sequentiell*	
Quellen	*Literaturdatenbanken & Metadatenbanken*	
Abdeckung	*Umfassend (kein Ausschluss)*	
Techniken	*Stichwortsuche (Suchstrings)*	*Rückwärtssuche*

Es wurde ein sequentieller Suchprozess verwendet und die Literatursuche zu Beginn des Review-Prozesses definiert. Im ersten Schritt wählten wir sechs bibliografische Datenbanken für die Literaturrecherche aus, darunter drei ohne spezifischen Forschungsschwerpunkt (z. B. SpringerLink) und die anderen mit einem Fokus auf Informationssystemen (z. B. AISeL):

- ACM Digital Library (ACM)
- Association for Information Systems eLibrary (AISeL)
- IEEE Xplore (IEEE)
- Litbaskets
- ScienceDirect
- SpringerLink

So konnten eine allgemeine Übersicht und ein breites Ergebnis erzielt werden, ohne den Fokus auf Informationssysteme zu verlieren. Im zweiten Schritt wurde der Suchstring definiert. Er umfasst vier Hauptkategorien, die für die Fragestellung relevant sind: den Kontext, in dem die Informationstechnologie verwendet wird, die Technologie selbst, Synonyme für Herausforderungen und die Verbindung zu digitalen Nomad*innen und Freiberufler*innen. Zu dieser Kategorie fügten wir den Begriff „Selbstständig“ als Synonym hinzu, da er häufig im Zusammenhang mit digitalen Nomad*innen und Freiberufler*innen verwendet wird. Um aus den ausgewählten Schlagwörtern einen Suchstring zusammenzustellen, wurden die logischen Operatoren AND und OR verwendet. Die Hauptkategorien wurden mit AND verbunden, während verwandte Begriffe innerhalb der Kategorien mit OR verbunden wurden. Die Suche wurde in jeder Datenbank mit denselben Stichwörtern durchgeführt. Einige der elektronischen Datenbanken haben jedoch unterschiedliche Vorgaben für Suchbegriffe. Daher mussten wir den Suchstring für zwei Datenbanken verfeinern, sichtbar in Tab. 4.

Mit diesen Suchstrings konnten so viele relevante Publikationen wie möglich gesammelt werden, um eine umfassende Auswertung der gefundenen Literatur zu ermöglichen. ScienceDirect erlaubt keine Suche mit mehr als acht booleschen Konnektoren und die Suche mit Litbaskets führte zu einem nicht zu umgehenden Fehler, weshalb diese beiden elektronischen Datenbanken aus der Liste der Datenbanken entfernt werden mussten. Das weitere Vorgehen beim systematischen Literaturreview wird in Abb. 2 gezeigt.

**Tab. 4:** Suchwörter bei der systematischen Literaturanalyse

Datenbank	Suchstring
ACM	AllField:(Virtual OR Collaboration OR Remote) AND AllField:(„Virtual Reality“ OR VR OR „Mixed Reality“) AND AllField:(Challenge OR Problem OR Risk) AND AllField:(„Digital Nomad“ OR Freiberufler*innen OR „Self Employed“)
IEEE	
AISeL	(Collaboration OR Virtual OR Remote) AND („Virtual Reality“ OR VR OR „Mixed Reality“) AND (Challenge OR Problem OR Risk) AND („Digital Nomad“ OR Freiberufler*innen OR „Self Employed“)
SpringerLink	

### B) Identifizierte Literaturquellen

- Alahuhta P, Nordbäck E, Sivunen A, Surakka T (2014) Fostering Team Creativity in Virtual Worlds. J Virtual Worlds Res 7:. 10.4101/jvwr.v7i3.7062
- Aridi A, Hayter CS, Radosevic S (2020) Windows of opportunities for catching up: an analysis of ICT sector development in Ukraine. J Technol Transf. 10.1007/s10961-020-09795-5
- Beno M (2019) Perspective on Slovakia’s Freelancers in Sharing Economy—Case Study. In: Advances in Intelligent Systems and Computing. Springer International Publishing, S 119–130
- Blomberg J, Karasti H (2013) Reflections on 25 Years of Ethnography in CSCW. Comput Support Coop Work 22:373–423. 10.1007/s10606-012-9183-1
- Bradley G (2006) Social Informatics—From Theory to Actions for the Good ICT Society. In: Social Informatics: An Information Society for all? In Remembrance of Rob Kling. Springer US, Boston, MA, S 383–394
- Broll W, Lindt I, Herbst I, et al. (2008) Toward next-gen mobile AR games. IEEE Comput Graph Appl 28:40–48. 10.1109/MCG.2008.85
- Curley J, Canning J, U EA, et al. (2019) VR/AR/MR for everyone! In: ACM SIGGRAPH 2019 Panels. ACM, New York, NY, USA, S 1–2
- Darnall BD, Krishnamurthy P, Tsuei J, et al. (2020) Self-Administered Skills-Based Virtual Reality Intervention for Chronic Pain: Randomized Controlled Pilot Study. JMIR Form Res 4:19. 10.2196/17293
- de Vreede T, Nguyen C, de Vreede G‑J, et al. (2013) A Theoretical Model of User Engagement in Crowdsourcing. In: Lecture Notes in Computer Science (including subseries Lecture Notes in Artificial Intelligence and Lecture Notes in Bioinformatics). S 94–109
- Giakoumis D, Votis K, Altsitsiadis E, et al. (2019) Smart, personalized and adaptive ICT solutions for active, healthy and productive ageing with enhanced workability. In: Proceedings of the 12th ACM International Conference on PErvasive Technologies Related to Assistive Environments. ACM, New York, NY, USA, S 442–447
- Jarvenpaa SL, Knoll K, Leidner DE (1997) Is anybody out there? Antecedents of trust in global virtual teams. J Manag Inf Syst 14:29–64. 10.1080/07421222.1998.11518185
- Kirkman BL, Mathieu JE (2005) The Dimensions and Antecedents of Team Virtuality. J Manage 31:700–718. 10.1177/0149206305279113
- Kishore R, McLean ER (2002) The Next Generation Enterprise: A CIO Perspective on the Vision, its Impacts, and Implementation Challenges. Inf Syst Front 4:121–138
- Kugler L (2021) The state of virtual reality hardware. Commun ACM 64:15–16. 10.1145/3441290
- Kugler L (2017) Why virtual reality will transform a workplace near you. Commun ACM 60:15–17. 10.1145/3105444
- Mesquita A, Camarinha AP, Lopes FC, Malta P (2020) What Will the Future of Work Look like for IS Professionals? The Picture of Portugal. In: IFIP Advances in Information and Communication Technology. pp 341–358
- Montealegre R, Cascio WF (2017) Technology-driven changes in work and employment. Commun ACM 60:60–67. 10.1145/3152422
- Patokorpi E (2006) Abductive Reasoning and ICT Enhanced Learning: Towards the Epistemology of Digital Nomads. In: The Information Society: Emerging Landscapes. Kluwer Academic Publishers, Boston, S 101–117
- Ratalewska M (2020) The Use of IT Tools in Small Businesses in Poland. Eur Bus Persp S 329–342 Springer, cham: Swizerland
- Regenbrecht H, Haller M, Hauber J, Billinghurst M (2006) Carpeno: interfacing remote collaborative virtual environments with table-top interaction. Virtual Real 10:95–107. 10.1007/s10055-006-0045-3
- Riemer K, Vehring N (2012) Virtual or vague? a literature review exposing conceptual differences in defining virtual organizations in IS research. Electron Mark 22:267–282. 10.1007/s12525-012-0094-2
- Schraefel MC, Ho J, Chignell M, Milton M (2000) Building virtual communities for research collaboration. In: Proceedings Academia/Industry Working Conference on Research Challenges ’00. Next Generation Enterprises: Virtual Organizations and Mobile/Pervasive Technologies. AIWORC’00. (Cat. No.PR00628). IEEE Comput. Soc, S 27–32
- Schultze U, Hiltz SR, Nardi B, et al. (2008) Using Synthetic Worlds for Work and Learning. Commun Assoc Inf Syst 22:. 10.17705/1CAIS.02219
- Schultze U, Rennecker J (2007) Refraining Online Games. In: Virtuality and Virtualization. Springer US, Boston, MA, S 335–351
- Wasko M, Teigland R, Leidner D, et al. (2011) Stepping into the Internet : New Ventures in Virtual Worlds Linked references are available on JSTOR for this article : Stepping into the Internet : New Ventures in Virtual Worlds. MIS Q 35:645–652

## Footnotes

1 https://spatial.io/ (abgerufen am 15. Juli 2021).

2 https://www.oculus.com/quest-2/ (abgerufen am 15. Juli 2021).
